# Changes in vascular function and correlation with cardiotoxicity in women with newly diagnosed breast cancer undergoing HER2-directed therapy with and without anthracycline/cyclophosphamide

**DOI:** 10.1093/ehjopen/oead130

**Published:** 2023-12-08

**Authors:** Antonious Hazim, Lara F Nhola, Vidur Kailash, Song Zhang, Nicole P Sandhu, Amir Lerman, Charles L Loprinzi, Kathryn J Ruddy, Hector R Villarraga, Bradley Lewis, Joerg Herrmann

**Affiliations:** Department of Oncology, Mayo Clinic, Rochester, MN, USA; Department of Cardiovascular Medicine, Mayo Clinic, Rochester, MN, USA; Department of Cardiovascular Medicine, Mayo Clinic, Rochester, MN, USA; Department of Cardiovascular Medicine, Mayo Clinic, Rochester, MN, USA; Department of Internal Medicine, Mayo Clinic, Rochester, MN, USA; Department of Cardiovascular Medicine, Mayo Clinic, Rochester, MN, USA; Department of Oncology, Mayo Clinic, Rochester, MN, USA; Department of Oncology, Mayo Clinic, Rochester, MN, USA; Department of Cardiovascular Medicine, Mayo Clinic, Rochester, MN, USA; Department of Biostatistics, Mayo Clinic, Rochester, MN, USA; Department of Cardiovascular Medicine, Mayo Clinic, Rochester, MN, USA

**Keywords:** Breast cancer, Cardiovascular risk, Endothelial function, HER2, Anthracycline

## Abstract

**Aims:**

The objective of this study was to assess the effect of HER2-directed therapy (HER2-Tx) on peripheral vasoreactivity and its correlation with cardiac function changes and the additive effects of anthracycline/cyclophosphamide (AC) therapy and baseline cardiovascular risk.

**Methods and results:**

Single-centre, prospective cohort study of women with newly diagnosed stage 1–3 HER2-positive breast cancer undergoing HER2-Tx +/− AC. All participants underwent baseline and 3-monthly evaluations with Endo-Peripheral Arterial Tonometry (Endo-PAT), vascular biomarkers [C-type natriuretic peptide (CNP) and neuregulin-1 beta (NRG-1β)], and echocardiography. Cardiotoxicity was defined as a decrease in the left ventricular ejection fraction (LVEF) of >10% to a value <53%. Of the 47 patients enrolled, 20 (43%) received AC in addition to HER2-Tx. Deterioration of reactive hyperaemia index (RHI) on Endo-PAT by ≥20% was more common in patients receiving HER-Tx plus AC than HER2-Tx alone (65% vs. 22%; *P* = 0.003). A decrease in CNP and log NRG-1β levels by 1 standard deviation did not differ significantly between the AC and non-AC groups (CNP: 20.0% vs. 7.4%; *P* = 0.20 and NRG-1β: 15% vs. 11%; *P* = 0.69) nor did GLS (35% vs. 37%; *P* = 0.89). Patients treated with AC had a significantly lower 3D LVEF than non-AC recipients as early as 3 months after exposure (mean 59.3% (SD 3) vs. 63.8% (SD 4); *P* = 0.02). Reactive hyperaemia index and GLS were the only parameters correlating with LVEF change.

**Conclusion:**

Combination therapy with AC, but not HER2-Tx alone, leads to a decline in peripheral vascular and cardiac function. Larger studies will need to define more precisely the causal correlation between vascular and cardiac function changes in cancer patients.

## Graphical Abstract

**Graphical abstract:**
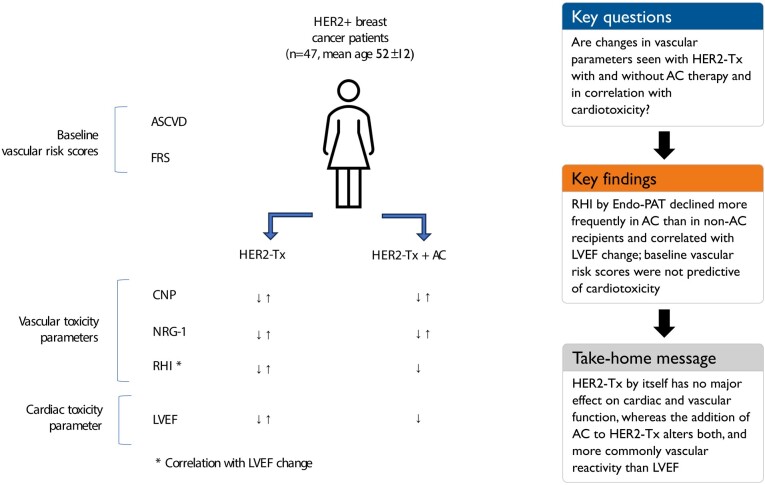

## Introduction

Human epidermal growth factor receptor 2 (HER2)-directed therapy (HER2-Tx) with drugs such as trastuzumab has revolutionized the treatment of HER2-positive breast cancer but with a black box warning of cardiotoxicity.^1^ Most of the time, this is in form of a decline in cardiac function whereas heart failure is rare (∼15–20% and <5% of patients, respectively). As serendipitous as the occurrence of cardiotoxicity was the discovery that HER-2 links to a stress signalling pathway in the heart, which could be stimulated, for instance, by anthracycline exposure, ischaemia, or hypertension. The ‘multiple hit theory’ conveyed that trastuzumab adds to any pre-existing cardiovascular (CV) disease (CVD) and risk factors in reducing the cardiac reserve to such a degree that it can become clinically evident.^2^ If so, integrative indices of CV risk such as Framingham Risk Score (FRS) and the American College of Cardiology (ACC)/American Heart Association (AHA) atherosclerotic cardiovascular disease (ASCVD) risk score might be of value.^3^ Furthermore, vasoreactivity/endothelial function assessment might be useful, reflecting the impact of CV risk factors on the CV system.^4,5^

Of further note in this context is the fact that the molecular pathway targeted by HER2 inhibitors involves Neuregulin-1β (NRG-1β), which is produced by the endothelium in the myocardial microcirculation and leads to activation of the HER-2 signalling cascade in cardiomyocytes.^6^ C-type natriuretic peptide (CNP) is a protein produced by both endothelial cells and cardiomyocytes, in distinction from B-type natriuretic peptide (BNP) and cardiac troponin (cTn), which are produced solely by cardiomyocytes.^7–13^ Together, these biomarkers could potentially provide a differential reflection of the endothelial–myocardial interaction so unique to HER-2-induced cardiomyopathy (vascular hypothesis of cardiotoxicity).^14^ A third unique biomarker in this context is vasoreactivity, especially endothelial function, with the reactive hyperaemia index (RHI) assessed by Endo-Peripheral Arterial Tonometry (Endo-PAT) as one of its measures.^4,5,15–17^ Two prior studies noted a decline in peripheral endothelial/vascular function in breast cancer patients undergoing aromatase inhibitor therapy.^18,19^ Certainly, the outlined approaches provide uniquely different and possibly complementary information from those currently favoured modes of cardiotoxicity surveillance, namely cTn, BNP, and speckle tracking (strain) echocardiographic imaging,^20–25^

The objective of this study was to assess the effect of HER2-Tx on peripheral vasoreactivity and its correlation with cardiac function changes as well as the additive effects of anthracycline and cyclophosphamide therapy (henceforth referred to as AC) and baseline CV risk on vascular and cardiac function in HER2-positive breast patients. Regarding the first aspect, changes in vascular function or vascular biomarkers may or may not occur with cancer therapies and may or may not correlate with (or even precede) cardiac function changes. Regarding the second aspect, based on the multiple hit theory one may assume greater CV changes (i) in HER2-Tx patients receiving AC therapy (additive risk) and (ii) in patients with greater baseline CV risk factor or CVD burden, evident in a higher FRS and the ASCVD risk score and abnormal peripheral vasoreactivity (RHI) by Endo-PAT (unmasked risk).

## Methods

### Study population

This single-centre, prospective, cohort study was approved by the Mayo Clinic Institutional Review Board, and informed consent was obtained from all participants. Participants were recruited through the Breast Diagnostic and Cancer Clinics at the Mayo Clinic in Rochester, Minnesota from 2016 to 2017. Women >18 years of age with newly diagnosed stage 1–3 HER-2 positive breast cancer who planned to undergo HER2-Tx with or without dose-dense AC were eligible for this study. Women were excluded if they had a left ventricular ejection fraction (LVEF) < 50% at the time of enrolment. Patients were followed prospectively approximately every 3 months for the duration of HER2-Tx.

### Study protocol

After enrollment, consented patients underwent cardiac assessments at seven time points: pre-therapy (baseline), pre-AC therapy (completed only for those patients undergoing AC therapy), pre-HER2-Tx, and then 3 months, 6 months, 9 months, and 12 months after initiation of HER2-Tx. At enrollment, a health profile was created to record age, menopausal status, breast cancer details, CVD, and CV risk factors, including smoking and family history.

Data metrics collected at each time point included: clinical variables: vital signs, toxicities, and medication adjustments. Lipid profile was measured at baseline only. Transthoracic echocardiogram (TTE), electrocardiogram (ECG), Endo-Peripheral Arterial Tonometry (Endo-PAT), and vascular biomarkers (CNP and NRG-1β) were obtained at each time point.

TTE imaging included serial 2D- and 3D-TTE with speckle tracking imaging. For 2D TTE, parasternal and apical views were obtained by a registered diagnostic cardiac sonographer following the current American Society of Echocardiography guidelines.^26^ A 3D full volume dataset of the ventricle was acquired using a gated acquisition with sector size, depth, and the number of heart beats optimized to obtain the highest possible volume rates. The LV volume and EF were measured using TomTec (Unterschleissheim, Germany) software for 3D acquisition and by manual contouring of the four- and two-chamber views for 2D acquisition. Strain measurements were performed offline with TomTec software for 2D and 3D acquisitions. Reactive hyperaemia index was measured by use the FDA-approved EndoPAT2000 device (Zoll Itamar, Atlanta, GA, USA). NRG-1β and CNP were measured by use of commercial ELISAs (NRG-1β1, Thermo fisher, Waltham, MA, USA, product code EHNRG1, range 82.3–20 000 pg/mL) and (Human CNP/NPPC,Novus Biologicals, Centennial, CO, USA, product code NBP2-62165, sensitivity 0.2 pmol/L).

### Outcome

The primary parameter of this study was an analysis of LVEF, and the primary study endpoint to be evaluated was the incidence of HER2-Tx-induced cardiotoxicity, defined according to the joint consensus from the American Society of Echocardiography and European Association of Cardiovascular Imaging as a decrease in the LVEF of >10%, to a value <53% at any follow-up time point (which was the leading definition at the time of conceptualization and initiation of this study). The secondary study parameters to be documented and analysed were CNP and NRG-1β levels, and the following echocardiographic parameters: left ventricular end-systolic and end-diastolic function and global longitudinal strain (GLS). Framingham Risk Score and ASCVD risk score were calculated from data collected at the beginning of the study. RHI scores were collected at baseline and at each follow-up visit.

### Data analysis

Continuous variables were presented as means (standard deviation) or medians (25th quartile–75th quartile). Categorial variables were presented as frequencies (percent). Two-sample *t*-tests for continuous variables and chi square tests for categorical variables were used to test group comparisons. NRG-1β levels were found to be skewed, so a log transformation was applied, and all results in this article reflect the log transformed NRG-1β values, unless specified otherwise. RHI deterioration was defined as a 20% reduction from baseline RHI to any available follow-up RHI assessment, in keeping with a prior study.^19^ Decreases in CNP, NRG-1β, and GLS levels were defined as a greater than or equal to 1 standard deviation reduction from baseline. Multiple univariate logistic regressions were used to assess the relationship of RHI deterioration, CNP decrease, log NRG-1β decrease, and GLS decrease with baseline covariates. Additionally, plots of the mean as a point estimate with accompanying standard error bars were made to visually demonstrate the change in values over time. A two-sample *t*-test was performed to assess the differences between groups at each visit. To assess the correlations between biomarkers, LVEF, and RHI, repeated measures correlations (RMC) were computed which represent the within-patient relationship among paired measures over multiple time points. Lastly, cumulative incidence curves were built to assess the declines in a time to event analysis where time was the respective visit. It was assumed that if a patient was missing a visit that they did not have a decline at that timepoint. Differences between groups were tested using a log-rank test. All *P*-values are two-sided interpreted with a 0.05 type I error rate. Analyses were conducted using R version 4.2.2 (R Foundation for Statistical Computing, Vienna, Austria).

## Results

### Patient, malignancy- and CV risk factor/disease-related characteristics

Our study cohort consisted of 47 women with newly diagnosed HER2-positive breast cancer. Details regarding the study population including treatment regimens and dosing are summarized in *Table 1*. Forty-five patients had newly diagnosed invasive ductal carcinoma; two patients had newly diagnosed invasive lobular carcinoma. A total of 20 patients (43%) received four cycles of AC with a median cumulative doxorubicin dose of 238 mg/m^2^ (range: 201–246 mg/m^2^). There were no significant differences in other therapies, including aromatase inhibitor use between those who did and those who did not receive AC therapy. No patient had a history of coronary artery disease, myocardial infarction, heart failure, chronic obstructive pulmonary disease, cardiomyopathy, chest radiation, or percutaneous coronary intervention or coronary artery bypass graft. The most common CV risk factors were hyperlipidaemia (21%) and hypertension (17%) (*Table 2*). The median ASCVD was 2.0 (0.6–3.7), and the median FRS was 1.0 (0.2–2.0). No patient had either a high FRS (>20%), or a high ASCVD score (>20%) and neither score was predictive of cardiotoxicity or correlated with LVEF decline.

**Table 1:** Breast cancer characteristics and treatment, all patients underwent HER2-directed therapy (HER2-Tx), with or without anthracycline/cyclophosphamide (AC)

Characteristics	All HER2-Tx (*n* = 47)	AC recipients (*N* = 20)	Non-AC recipients (*N* = 27)	*P*-value
**Cancer Type**				
Invasive ductal carcinoma, *n* (%)	45 (96)	20 (100)	25 (93)	0.11
Invasive lobular carcinoma, *n* (%)	2 (0.4)	0 (0)	2 (0.7)	0.11
Lymph node involvement, *n* (%)	12 (26)	6 (30)	6 (13)	0.28
**Neoadjuvant Regimen**				
Four cycle therapy, *n* (%)	35 (75)	19 (95)	16 (59)	**0**.**01**
Six cycle therapy, *n* (%)	12 (26)	1 (5)	11 (41)	**0**.**01**
Dual HER2-directed therapy, *n* (%)	38 (81)	18 (90)	20 (74)	0.2
Mono HER2-directed therapy, *n* (%)	9 (19)	2 (10)	7 (26)	0.2
Herceptin loading dose (mg)	564 (47)	570 (20)	560 (27)	0.2
Herceptin subsequent dose (mg)	410 (47)	410 (20)	410 (27)	0.3
Pertuzumab loading dose (mg)	840 (38)	840 (18)	840 (20)	0.2
Pertuzumab subsequent dose (mg)	420 (38)	420 (18)	420 (20)	0.3
Docetaxel dose (mg/m^2^)	150 (17)	155 (11)	140 (6)	0.07
Paclitaxel dose (mg/m^2^)	162 (22)	157.5 (14)	170 (8)	0.2
Carboplatin dose (mg)	725 (8)	—	725 (8)	—
**Anthracycline Regimen**				
Four cycle therapy, *n* (%)	20 (43)	20 (100)	—	—
Cyclophosphamide total dose (mg/m^2^)	1150 (20)	1150 (20)	—	—
Doxorubicin cumulative dose (mg/m^2^)	238 (20)	238 (20)	—	—
**Breast Surgery, *n* (%)**	**47** (**100)**	20 (100)	27 (100)	—
Lumpectomy, *n* (%)	11 (23)	5 (25)	6 (22)	0.8
Mastectomy-unilateral, *n* (%)	13 (28)	3 (15)	10 (37)	0.1
Mastectomy-bilateral, *n* (%)	23 (49)	12 (60)	11 (41)	0.2
**Adjuvant Radiation Therapy, *n* (%)**	**30** (**64)**	14 (70)	16 (59)	0.5
Number of fractions	25	25	25	0.1
Dose delivered (cGY)	5000	5000	5000	0.5
Proton bean technique, *n* (%)	14 (30)	6 (30)	8 (30)	0.9
Multi-field photon technique, *n* (%)	16 (34)	8 (40)	8 (30)	0.6
**Adjuvant HER-2 Inhibitor Therapy, *n* (%)**	**47** (**100)**	20 (100)	27 (100)	—
Herceptin loading dose (mg)	615 (29)	619 (18)	607 (11)	0.4
Herceptin subsequent dose (mg)	457 (47)	464 (20)	451 (27)	0.2
Pertuzumab dose (mg)	420 (4)	420 (2)	420 (2)	0.3
Taxol dose (mg/m^2^)	125 (1)	—	125 (1)	—
**Hormone Status**				
HER2-positive, *n* (%)	47 (100)	20 (100)	27 (100)	—
Oestrogen receptor +/progesterone receptor +, *n* (%)	23 (49)	10 (50)	13 (11)	0.9
Oestrogen receptor +/progesterone receptor −, *n* (%)	7 (15)	4 (20)	3 (48)	0.4
Oestrogen receptor −/progesterone receptor +, *n* (%)	1 (2)	1 (5)	0 (0)	0.2
Oestrogen receptor −/progesterone receptor −, *n* (%)	16 (34)	5 (25)	11 (41)	0.3
**Adjuvant Endocrine Therapy, *n* (%)**	**27** (**57)**	13 (65)	14 (52)	0.4
Tamoxifen, *n* (%)	11 (23)	6 (30)	5 (19)	0.4
Aromatase inhibitor, *n* (%)	16 (34)	7 (35)	9 (33)	0.2

Drug doses expressed as mean (standard deviation, SD), otherwise number (%).

Bold indicates statistical significance.

**Table 2:** Cardiovascular characteristics and medications

Characteristics	All HER2-Tx (N = 47)	AC recipients (*N* = 20)	Non-AC recipients (*N* = 27)	*P*-value
**Age (years)**	52 (12)	48 (12)	54 (13)	0.07
**White, *n* (%)**	46 (98)	20 (100)	26 (96)	0.2
**Cardiovascular Risk Factors**				
** **Current smoker, *n* (%)	2 (4)	1 (5)	1 (4)	0.4
** **Former smoker, *n* (%)	7 (15)	3 (15)	4 (15)	0.5
** **Hypertension, *n* (%)	8 (17)	4 (20)	4 (15)	0.3
** **Hyperlipidaemia, *n* (%)	10 (21)	4 (20)	6 (22)	0.4
** **Diabetes mellitus, *n* (%)	2 (4)	0 (0)	2 (7)	0.2
** **Family history of premature CAD, *n* (%)	2 (4)	1 (5)	1 (4)	0.4
**Cardiovascular Diseases**				
** **Valvular disease, *n* (%)	2 (4)	1 (5)	1 (4)	0.4
** **Heart failure, *n* (%)	0 (0)	0 (0)	0 (0)	0
** **Atrial fibrillation, *n* (%)	0 (0)	0 (0)	0 (0)	0
** **Peripheral arterial disease, *n* (%)	0 (0)	0 (0)	0 (0)	0
** **Coronary artery disease, *n* (%)	0 (0)	0 (0)	0 (0)	0
** **ASCVD risk score (SD)	2.8 (3)	2 (2)	3(3)	0.1
** **Framingham risk score (SD)	1.3 (1)	1 (1)	1.4 (1)	0.3
**Baseline Measures**				
Body mass index (kg/m^2^), mean (SD)	28 (7)	28 (6)	27 (8)	0.2
Systolic blood pressure (mmHg), mean (SD)	112 (19)	114 (22)	110 (19)	0.5
Diastolic blood pressure (mmHg), mean (SD)	70 (12)	72 (11)	68(8)	0.4
** **Heart rate (beats/min), mean (SD)	71 (15)	66 (15)	68 (17)	0.2
** **LVEF (%), mean (SD)	63 (4)	63 (4)	63 (3)	0.89
**Baseline Medications**				
** **Aspirin, *n* (%)	7 (15)	3 (15)	4 (15)	0.5
** **Beta blockers, *n* (%)	4 (9)	2 (10)	2 (8)	0.4
Angiotensin converting enzyme (ACE) inhibitors and angiotensin receptor blockers (ARB), *n* (%)	6 (13)	2 (10)	4 (15)	0.3
** **Calcium channel blockers, *n* (%)	2 (4)	0 (10)	2 (8)	0.1
** **Statins, *n* (%)	6 (13)	2 (10)	4 (15)	0.3
**CV Drug Modification, *n***				
** **Visit 2	2	2 (carvedilol)	1 (started on carvedilol, lisinopril increased)	
** **Visit 3	2	0	2 (started on carvedilol, lisinopril increased; started on lisinopril)	
** **Visit 4	0	0	0	
** **Visit 5	1	1 (carvedilol)	0	
** **Visit 6	0	0	0	
** **Visit 7	0	0	N/A	

All patients underwent HER2-directed therapy (HER2-Tx), with or without anthracycline/cyclophosphamide (AC).

### Peripheral vasoreactivity testing

**Figure 1:**
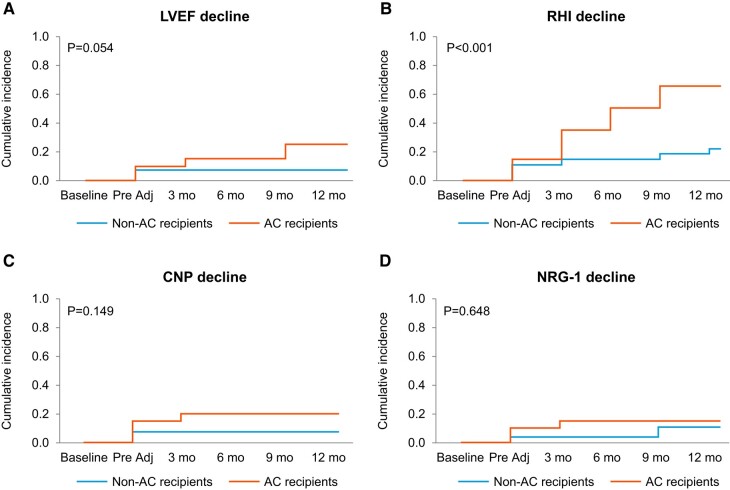
Cumulative incidence of decline in left ventricular ejection fraction (LVEF, *A*), reactive hyperaemia index (RHI, *B*), C-type natriuretic peptide (CNP, *C*) serum levels, and neuregulin-1 beta (NRG-1β, D) serum levels by stated criteria in patients with and without anthracycline–cyclophosphamide (AC) in addition to HER2-Tx.

**Figure 2:**
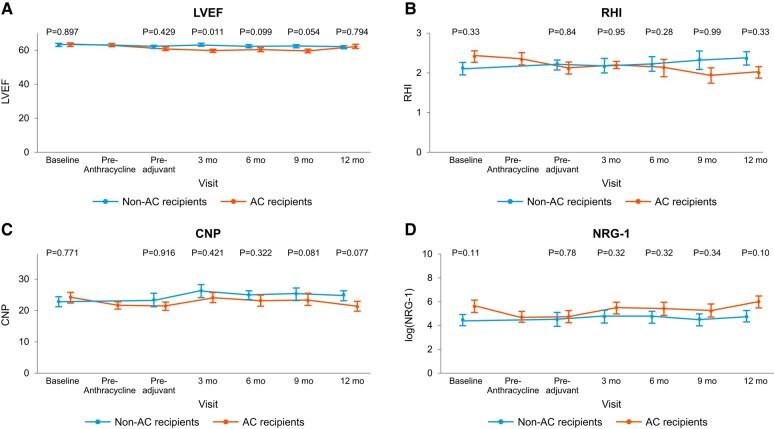
Left ventricular ejection fraction (LVEF, *A*), reactive hyperaemia index (RHI, *B*), C-type natriuretic peptide (CNP, *C*) serum levels, and neuregulin-1 beta (NRG-1β, *D*) serum levels in patients with and without anthracycline–cyclophosphamide (AC) in addition to HER2-Tx.

Thirty-eight of the 47 patients (81%) underwent endothelial function testing at baseline, 17 (50%) of whom subsequently received AC therapy. These 38 patients underwent an average of five (SD 1.5) Endo-PAT tests over time (145 total RHI assessments). Ten of the 38 patients undergoing Endo-PAT testing (26.3%) had an abnormal RHI (≤1.67) at baseline. RHI deterioration by 20% or more was more common among AC recipients (mean 65% vs. 22%; *P* = 0.003) and became evident after AC exposure (*Figure 1*). Anthracycline use (odds ratio: 6.5; 95% confidence interval: 1.87–25.4; *P* = 0.004) and baseline RHI (odds ratio: 27.6; 95% confidence interval: 4.7—340.5; *P* = 0.002) were independent predictors of RHI deterioration (Table 3). The absolute RHI values showed a decline immediately after AC therapy and over time while on HER-2 directed therapy (*Figure 2*). No significant differences in RHI values were observed between the binary groups of those who did vs. those who did not experience cardiotoxicity (see Supplementary material online, *Figures S1* and *S2*).

### Vascular biomarkers

Forty-five of the 47 patients (96%) had baseline CNP and NRG-1β levels measured and had a total of 253 measurements of CNP and NRG-1β values over time [mean of 5 (SD 1.3) CNP and NRG-1β levels, each]. Decreases in CNP and NRG-1β levels were numerically, but not statistically, more common in AC recipients (CNP: 20% vs. 7.4%; *P* = 0.20 and NRG-1β: 15% vs. 11%; *P* = 0.69, *Figure 1*). At 3, 6, 9, and 12 months posttherapy, patients who received AC had lower CNP levels and log NRG-1β levels, though these differences were not significant (*Figure 2*). A trend was observed for lower NRG-1β levels at baseline and early on among those patients who developed cardiotoxicity (see Supplementary material online, *Figures S1* and *S2*). Conversely, higher CNP levels at baseline were noted in the cardiotoxicity group and baseline CNP was identified as a potential predictor of cardiotoxicity (*Table 3*).

**Table 3:** Odds ratios and 95% confidence intervals from univariate logistic regression for reactive hyperaemia index (RHI) deterioration, C-type natriuretic peptide (CNP) decrease, neuregulin (NRG) decrease, and cardiotoxicity

	RHI deterioration	CNP decline	NRG decrease	GLS decrease	Cardiotoxicity
**Age**	1.00 (0.95–1.05)	0.98 (0.91–1.05)	1.10 (1.01–1.22)*	1.01 (0.97–1.07)	0.97 (0.90–1.03)
**Baseline RHI**	27.6 (4.7–340)*	0.47 (0.07–2.10)	0.37 (0.04–1.43)	1.16 (0.42–3.24)	0.50 (0.09–1.99)
**Baseline CNP**	1.02 (0.95–1.11)	1.14 (1.02–1.30)*	1.04 (0.93–1.15)	0.99 (0.90–1.07)	1.11 (1.01–1.26)*
**Baseline log NRG-1β**	0.99 (0.76–1.28)	1.22 (0.83–1.87)	1.04 (0.71–1.53)	0.88 (0.67–1.15)	0.76 (0.48–1.12)
**Baseline GLS**	1.23 (0.90–1.73)	0.85 (0.53–1.33)	1.04 (0.71–1.53)	1.39 (1.00–2.01)	1.30 (0.85–2.12)
**Anthracycline**	6.50 (1.87–25.4)*	3.13 (0.54–24.5)	1.41 (0.24–8.46)	0.92 (0.27–3.05)	4.17 (0.79–31.7)
**ASCVD score**	0.95 (0.74–1.19)	0.74 (0.30–1.16)	0.25 (0.002–1.08)	0.82 (0.59–1.05)	0.94 (0.60–1.28)
**Framingham risk score**	0.74 (0.41–1.25)	0.54 (0.13–1.32)	1.60 (0.50–5.47)	0.67 (0.34–1.18)	0.63 (0.21–1.38)

^*^ *P* < 0.05.

### Transthoracic echocardiogram and electrocardiogram monitoring

A total of 45 patients underwent LVEF assessment at baseline and had a mean of 5 echocardiograms (SD 1.5). A summary of the echocardiogram findings is provided in *Table 4*. Patients treated with AC had a significantly lower LVEF than non-AC recipients as early as 3 months after exposure: 3D (General Electrics, GE) EF [mean 59.3% (SD 3) vs. 63.8% (SD 4); *P* = 0.02]. At that time point end-systolic volume (ESV) was higher in the AC group, while there was no difference in end-diastolic volume (EDV) or blood pressure between the groups. A significantly higher EDV was seen in the AC group at the 6 months follow-up, but still no difference in blood pressure. Changes in ESV and EDV, compared to baseline, were only seen in recipients of AC therapy. In those treated with AC, 7 (14.9%) patients had an LVEF decrease of >10%, to a value <53% over the follow-up period (*Table 4*). Abnormal GLS values (−18% or higher) were noted in 4 (20%) patients in the AC and in 5 (18.5%) patients in the non-AC group (*P* = 0.91). There was no difference in the rate of change in GLS by 1 SD between the AC and the non-AC group (35% vs. 37%; *P* = 0.89). For correlation of GLS with LVEF, please see below. No significant changes in ECGs were noted (i) between the groups or (ii) compared to baseline.

**Table 4:** Echocardiographic data among cohorts

Variable	Baseline Non-AC	Baseline AC	Baseline Non-AC	Pre-AC AC	Pre-adjuvant Non-AC	Pre-adjuvant AC	3 months Non-AC	3 months AC	6 months Non-AC	6 months AC	9 months Non-AC	9 months AC	12 months Non-AC	12 months AC
**Vital signs**														
SBP (mmHg)	110.2 (19)	114.5 (22)	110.2 (19)	109.5 (21)	109.9 (19)	107.2 (17)	110.8 (21)	116.1 (18)	111 (19)	114.25 (8)	113.6 (19)	110.5 (19)	106.4 (17)	112 (13)
DBP (mmHg)	68.5 (8)	72.3 (11)	68.5 (8)	69.7 (13)	67.7 (10)	68.4 (10)	66.4 (12)	74.1 (14)	69 (5)	69 (5)	67.4 (5)	65.7 (3)	62.4 (5)	68 (6)
HR (beats/min)	68 (17)	66.8 (15)	68 (17)	71 (1)	71 (11)	72 (11)	67 (6)	68 (11)	63 (8)	66 (11)	65 (8)	72 (11)	64 (11)	61 (8)
**Echocardiographic parameters**														
**GLS (%)**	−20.5 (8)	−21.3 (2)	−20.5 (8)	−21.5 (2)	−21.4 (3)	−20.8 (2)	−21.8 (2)	−21.8 (2)	−21.4 (2)	−21.3 (2)	−21.2 (1)	−20.8 (2)	−21.3 (1)	−19.8 (1)
**Four-chamber volume**														
EDV (mL)	109.2 (17)	109.2 (17)	109.2 (17)	112.6 (21)	113.7 (19)	113.8 (15)	115.2 (22)	118.9* (15)	**109** (**20)**	123.8* (**18)**	111.5 (22)	120.6* (15)	117.4 (24)	121.3 (17)
ESV (mL)	40.5 (7)	40.5 (10)	40.5 (7)	43.6 (9)	42.2 (13)	43.6 (7)	42.5 (9)	46.4* (6)	**43.5** (**9)**	50.5* (**11)**	**42.7** (**1)**	51.8** **(8)**	45.2 (10)	48.3 (6)
EF (%)	62.9 (4)	63.2 (5)	62.9 (4)	61.4 (5)	63.1 (7)	61.6 (4)	62.9 (5)	60.9* (3)	61.3 (4)	59.2* (5)	**61.6** (**4)**	56.8** **(6)**	61.5 (3)	60 (2)
**Two-chamber volume**														
EDV (mL)	112.9 (21)	115.9 (20)	112.9 (21)	113.3 (21)	118.5 (22)	113.5 (15)	117.4 (23)	118.6 (19)	117 (16)	124.9 (21)	118 (24)	118.3 (19)	113.7 (24)	125.8 (11)
ESV (mL)	41.2 (10)	44.0 (8)	41.2 (10)	41.2 (9)	45.2 (13)	45.1 (10)	42.8 (8)	45.3 (5)	42.8 (8)	48.4 (10)	43.6 (12)	47.1 (10)	43.4 (9)	49.5 (5)
EF (%)	63.6 (4)	62.4 (5)	63.6 (4)	63.6 (4)	62.3 (5)	61.78 (5)	63.4 (3)	61.5 (4)	63.7 (4)	62.6 (4)	62.7 (4)	60.2 (5)	61.4 (5)	60.8 (3)
**Biplane volume**														
EDV (mL)	111.1 (18)	113.4 (18)	111.1 (18)	113.8 (19)	116.5 (19)	113.9 (15)	116.8 (22)	118.6 (15)	**113.4** (**17)**	125.1* (**20)**	115.5 (23)	120.4 (16)	116.4 (24)	123.5 (12)
ESV (mL)	40.9 (8)	42.8 (8)	40.9 (8)	42.5 (8)	43.8 (13)	43.6 (7)	42.8 (9)	45.7 (5)	44 (11)	48.5* (10)	43.6 (11)	49.9* (8)	44.2 (9)	49.8 (4)
EF (%)	63.3 (3)	62.2 (4)	63.3 (3)	62.6 (4)	62.8 (5)	61.6 (4)	**63.2** (**3)**	**61.1** (**3)**	62.5 (3)	61 (4)	62.5 (3)	60.4 (5)	61.9 (2)	59.8 (3)
**3D Philips volume**														
EDV (mL)	115.4 (16)	114.9 (14)	115.4 (16)	117.1 (17)	116.9 (19)	117.7 (14)	120.1 (22)	122.7 (15)	**115** (**20)**	**130.7^ (15)**	116 (18)	115.4 (12)	107.8 (25)	127.5 (19)
ESV (mL)	40.6 (7)	41.6 (7)	40.6 (7)	43 (7)	44.8 (11)	44.2* (6)	43.1 (9)	46.3* (6)	**43.5** (**8)**	50.6** **(5)**	46.8* (11)	48.6* (7)	**41.4** (**7)**	**49.5** (**5)**
EF (%)	65 (4)	64 (3)	65 (4)	63.3 (3)	62 (5)	62.4* (3)	**64.2** (**4)**	61.7* (**3)**	62.7* (**3)**	61.4* (**2)**	62.6* (3)	60.3** (3)	62.7* (2)	61* (2)
**3D GE volume**														
EDV (mL)	116.1 (17)	114.4(17)	116.1 (17)	117.5 (15)	117.3 (21)	118.8 (14)	117.5 (21)	119.9 (13)	**114.2** (**19)**	**131.9^ (19)**	117.8 (18)	119.5 (23)	112.4 (19)	117.3 (11)
ESV (mL)	42.2 (7)	42.2 (9)	42.2 (7)	43.3 (7)	45.3 (13)	46.6* (5)	**42.2** (**8)**	47.3* (**4)**	**43.1** (**8)**	52** **(9)**	**44.2** (**8)**	50.5* (**9)**	42.1 (8)	43 (3)
EF (%)	63.7 (3)	63.1 (4)	63.7 (3)	63.2 (3)	61.8 (5)	60.6 (4)	**63.8** (**4)**	59.9* (**3)**	**62.4** (**3)**	59.8* (**3)**	**62.1** (**3)**	57.5** **(6)**	62 (2)	60 (2)

All patients underwent HER2-directed therapy (HER2-Tx), with or without anthracycline/cyclophosphamide (AC).

SBP, systolic blood pressure; DBP, diastolic blood pressure; HR, heart rate; GLS, global longitudinal strain; EDV, end-diastolic volume; ESV, end-systolic volume; EF, ejection fraction.

Values are expressed as mean (SD).

* *P* < 0.05 compared to baseline, **P < 0.009 compared to baseline, Bold, *P* < 0.05 between cohort.

### Within-patient correlations

A repeated measures correlation (RMC) analysis was performed to determine whether the vascular biomarkers, RHI, GLS, and LVEF correlated with one another in those receiving AC therapy. Statistically significant correlations were found between CNP and log NRG-1β levels (RMC = 0.21; *P* = 0.03) but not log NRG-1β and RHI (RMC = −0.03; *P* = 0.78) or CNP and RHI (RMC = −0.07; *P* = 0.58). Log NRG-1β and CNP were not found to be significantly correlated with changes in LVEF (RMC = −0.07; *P* = 0.51 and RMC = −0.10; *P* = 0.34, respectively). The correlation between RHI and LVEF changes as a continuous variable (not binary outcome) was significant (RMC = 0.26; *P* = 0.03). GLS was found to be negatively correlated with LVEF (RMC = −0.31; *P* = 0.007), but not RHI, log NRG-1β, or CNP (RMC = 0.16; *P* = 0.25, RMC = 0.06; *P* = 0.58, and RMC = 0.10; *P* = 0.41, respectively).

### Adverse events

Supplementary material online, *Table S1* depicts patient clinical complaints noted at each visit. No significant difference was noted between the cohorts. Five patients, two in the AC and three in non-AC cohorts, required the initiation of carvedilol at follow-up visits, and two of the five patients also required up-titration of their lisinopril.

## Discussion

The key results of this study can be summarized as following: (i) the addition of AC therapy to trastuzumab but not trastuzumab therapy alone is associated with a change in peripheral vasoreactivity and (ii) peripheral vasoreactivity correlates with changes in LVEF.

The effects of cancer therapeutics on the circulatory system are broad and some of what is perceived as being cardiotoxicity may not be solely due to a direct effect on the myocardium, but may be due to concomitant effects on the vasculature, particularly the endothelium.^14,27,28^ One illustrating example where this might come into play is in patients undergoing HER-2-directed therapy. Endothelial cells produce, among other factors, NRG-1β, a ligand for HER-2 and the related stress activation pathway.^6^ Its reduction could henceforth blunt myocardial stress responses and precipitate more cardiac dysfunction. An injured or dysfunctional endothelium could be seen in patients with vascular disease and/or CV risk factors or exposure to cancer therapies. Specifically, prior studies found lower NRG-1 levels in patients with severe coronary artery disease (CAD) and those developing cardiotoxicity with trastuzumab.^29,30^ In the current study, we likewise noted lower NRG-1β values in patients who developed a decline in LVEF over the full course of trastuzumab therapy. In distinction to the prior study, however, NRG-1β levels were already lower at baseline in those who developed cardiotoxicity on therapy in the current study. Furthermore, an extreme range of NRG-1β values were seen necessitating the use of log transformation. Statistical significance was not reached and cutoffs could not be defined in this setting. A much larger study size is needed to address these aspects further.

Akin to NRG-1β, CNP has been linked to crosstalk functions across various cells in the heart, and henceforth coordinating and preserving coronary vasoreactivity, cardiac function, and cardiac structure.^31^ Clinical studies have indicated increasing levels of CNP with worsening degrees of heart failure (HF),^32,33^ but such correlations have not been confirmed by others.^34^ Ischaemic burden (±haemodynamic load) may influence CNP levels,^35^ and CNP levels may reflect at high-CV risk.^36^ Indeed, CNP has been considered a vascular marker as much as, if not more than, a cardiac marker. It is produced by endothelial cells, in particular upon cytokine stimulation.^37^ This may explain high levels under conditions of septic shock and other chronic inflammatory conditions.^38^ In the current study, we did not notice an increase in CNP levels; if anything, levels were lower in patients receiving AC therapy. CNP levels, however, were significantly higher at baseline in patients who developed a decline in cardiac function with cancer therapy, and baseline CNP was identified as a predictor of cardiotoxicity in this study. Further studies will be needed to define the optimal cutoff for baseline risk stratification.

Vascular function has been studied in several cohorts of cancer patients and includes prior work from our group in breast cancer patients.^19^ These patients, however, underwent aromatase inhibitor therapy only; those with chemotherapy or HER-2-directed therapy were excluded.^19^ The current study henceforth complements this prior study and, using the same definition for RHI change, it likewise shows a greater proportion of patients with a decline in RHI in patients after AC treatment. Conversely, there was no change in patients who underwent trastuzumab therapy only. Thus, one may conclude that not all cancer therapies affect vascular function, but aromatase inhibitors and AC therapy do. Supporting the current findings is the observation of impaired peripheral vasoreactivity/endothelial health in survivors of childhood acute lymphoid leukaemia or other haematological malignancies, a large proportion of which having been exposed to anthracyclines.^39–41^ These studies did not comment specifically on a concomitantly low cardiac function in these patients. In the current study, RHI did show a correlation with LVEF changes though not with the label of cardiotoxicity. This is likely explained by the binary nature of the second read-out within the confinement of small study numbers, i.e. stratification of patients into groups with limited numbers, which are then compared, rather than the comparison of all available RHI and LVEF values as in the repeated measures approach. Future work in a larger cohort will be required to further define the correlation and address what has been deemed as ‘the vascular hypothesis of cardiotoxicity’.^14^

While this was a prospective cohort study, which by design addresses several concerns associated with retrospective analyses, several limitations need to be considered. The main one is the small study size, limiting power to truly reject negative findings. On the flip side, results that reached significant levels are worth taking note of, even though they still need validation in larger and more diverse cohorts. The second limitation is that of a single-centre setting and results need to be reproduced in a larger and/or more diverse cohort, e.g. in lymphoma patients. This would also allow assessment of whether any observations pertain to male patients and whether they are due to anthracyclines or to other medications. Herein, we cannot exclude a combined or concomitant effect of cyclophosphamide and anthracyclines. However, it is in general difficult to define the nature of the culprit agent in cancer patients unless it is strictly one mode of therapy as in the subset of patients undergoing HER-2-directed therapy alone.

In conclusion, this study showed that HER-2-directed therapy alone has no major effect on vascular reactivity and vascular biomarkers, but the addition of AC can lead to a deterioration in vascular and cardiac function. RHI correlates with LVEF changes overall, and larger studies will be needed to define more precisely the correlation between vascular and cardiac function changes in cancer patients, and vascular function analysis as a window to cardiotoxicity risk assessment and reduction.

## Supplementary Material

oead130_Supplementary_Data

## Data Availability

The datasets generated and/or analysed during this study are available from the corresponding author on reasonable request. The study protocol and other related documents can also be made available to others on request. The corresponding time frame shall be with publication and can be shared as an electronic file after explicit approval of the study investigators and a signed data usage agreement between the participating institutions.

## Lead author biography

Antonious Hazim, M.D. is a hematology and medical oncology fellow at Mayo Clinic in Rochester, Minnesota. He is interested in early cancer therapeutics, remote patient monitoring, and thoracic malignancies.
