# Supplementary material for: Changes in vascular function and correlation with cardiotoxicity in women with newly diagnosed breast cancer undergoing HER2-directed therapy with and without anthracycline/cyclophosphamide
Source: Eur Heart J Open. 2023 Dec 8;4(1):oead130. doi: 10.1093/ehjopen/oead130 (PMC10794877; doi:10.1093/ehjopen/oead130)

**Supplemental Material**

**Supplemental Table 1.** Adverse events potentially indicative of heart failure.

| Characteristic | Total ( N=47) | Anthracycline recipients (n=20) | Non-anthracycline recipients (n=27) | P value |
| --- | --- | --- | --- | --- |
| Baseline |  |  |  |  |
| SOB | 0 (0) | 0 | 0 | 0 |
| Fatigue/weakness | 0 (0) | 0 | 2 | 0.21 |
| Chest Pain | 0 (0) | 0 | 0 | 0 |
| Palpitations | 0 (0) | 0 | 0 | 0 |
| Peripheral edema/ascites | 0 (0) | 0 | 0 | 0 |
| Sudden weight gain | 0 (0) | 0 | 0 | 0 |
| Anorexia and early satiety, weight loss | 0 (0) | 0 | 0 | 0 |
| Pre-Adjuvant |  |  |  |  |
| SOB | 9 (19) | 3 | 6 | 0.53 |
| Fatigue/weakness | 24 (51) | 10 | 14 | 0.9 |
| Chest Pain | 1 (2) | 0 | 1 | 0.38 |
| Palpitations | 1 (2) | 0 | 1 | 0.38 |
| Peripheral edema/ascites | 0 (0) | 0 | 0 | 0 |
| Sudden weight gain | 0 (0) | 0 | 0 | 0 |
| Anorexia and early satiety, weight loss | 1 (2) | 1 | 0 | 0.38 |
| 3 months |  |  |  |  |
| SOB | 2 (4) | 0 | 2 | 0.21 |
| Fatigue/weakness | 15 (32) | 7 | 8 | 0.7 |
| Chest Pain | 0 (0) | 0 | 0 | 0 |
| Palpitations | 2 (4) | 2 | 0 | 0.93 |
| Peripheral edema/ascites | 3 (6) | 1 | 2 | 0.74 |
| Sudden weight gain | 1 (2) | 0 | 1 | 0.38 |
| Anorexia and early satiety, weight loss | 1 (2) | 1 | 0 | 0.24 |
| 6 months |  |  |  |  |
| SOB | 6 (13) | 3 | 3 | 0.69 |
| Fatigue/weakness | 17 (36) | 8 | 9 | 0.64 |
| Chest Pain | 1 (2) | 1 | 0 | 0.24 |
| Palpitations | 2 (4) | 1 | 1 | 0.83 |
| Peripheral edema/ascites | 1 (2) | 0 | 1 | 0.38 |
| Sudden weight gain | 0 (0) | 0 | 0 | 0 |
| Anorexia and early satiety, weight loss | 0 (0) | 0 | 0 | 0 |
| 9 months |  |  |  |  |
| SOB | 5 (11) | 2 | 3 | 0.9 |
| Fatigue/weakness | 11 (23) | 2 | 9 | 0.06 |
| Chest Pain | 1 (2) | 1 | 0 | 0.24 |
| Palpitations | 2 (4) | 1 | 1 | 0.83 |
| Peripheral edema/ascites | 1 (2) | 0 | 1 | 0.38 |
| Sudden weight gain | 0 (0) | 0 | 0 | 0 |
| Anorexia and early satiety, weight loss | 0 (0) | 0 | 0 |  |
| 12 months |  |  |  |  |
| SOB | 7 (15) | 3 | 4 | 0.99 |
| Fatigue/weakness | 12 (26) | 3 | 9 | 0.15 |
| Chest Pain | 2 (4) | 1 | 1 | 0.83 |
| Palpitations | 3 (6) | 2 | 1 | 0.38 |
| Peripheral edema/ascites | 1 (2) | 0 | 1 | 0.38 |
| Sudden weight gain | 1 (2) | 0 | 1 | 0.38 |
| Anorexia and early satiety, weight loss | 0 (0) | 0 | 0 | 0 |

**Supplemental Figures**

**Figure 1.** Cumulative incidence of decline in reactive hyperemia index (RHI, panel A), C-type natriuretic peptide (CNP, panel B) serum levels, and neuregulin-1 (NRG1, panel C) serum levels by stated criteria in patients with and without cardiotoxicity.

**Figure 2.** Left ventricular ejection fraction (LVEF, panel A), reactive hyperemia index (RHI, panel B), C-type natriuretic peptide (CNP, panel C) serum levels, and neuregulin-1 (NRG1, panel D) serum levels in patients with and without cardiotoxicity.


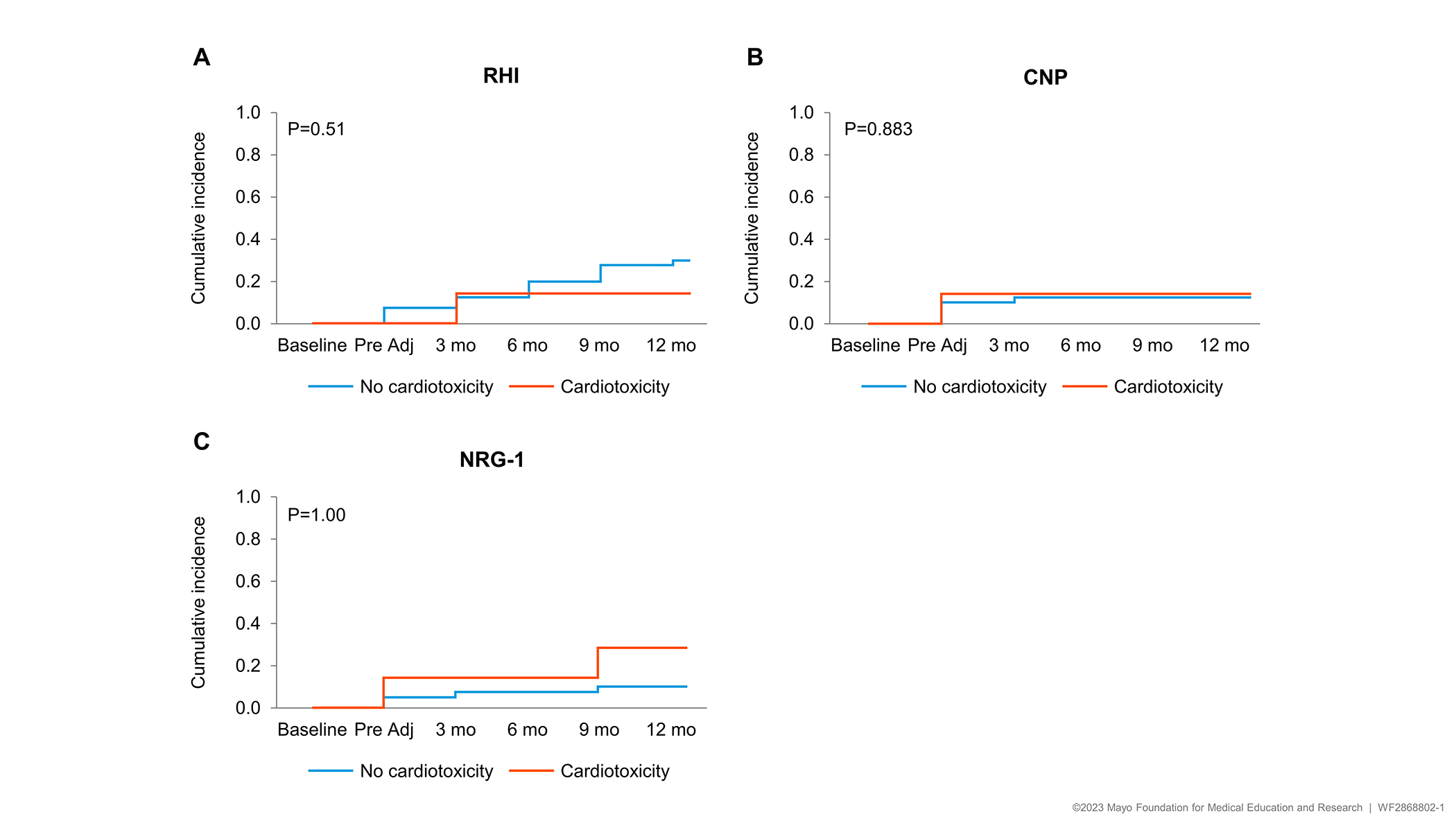


A


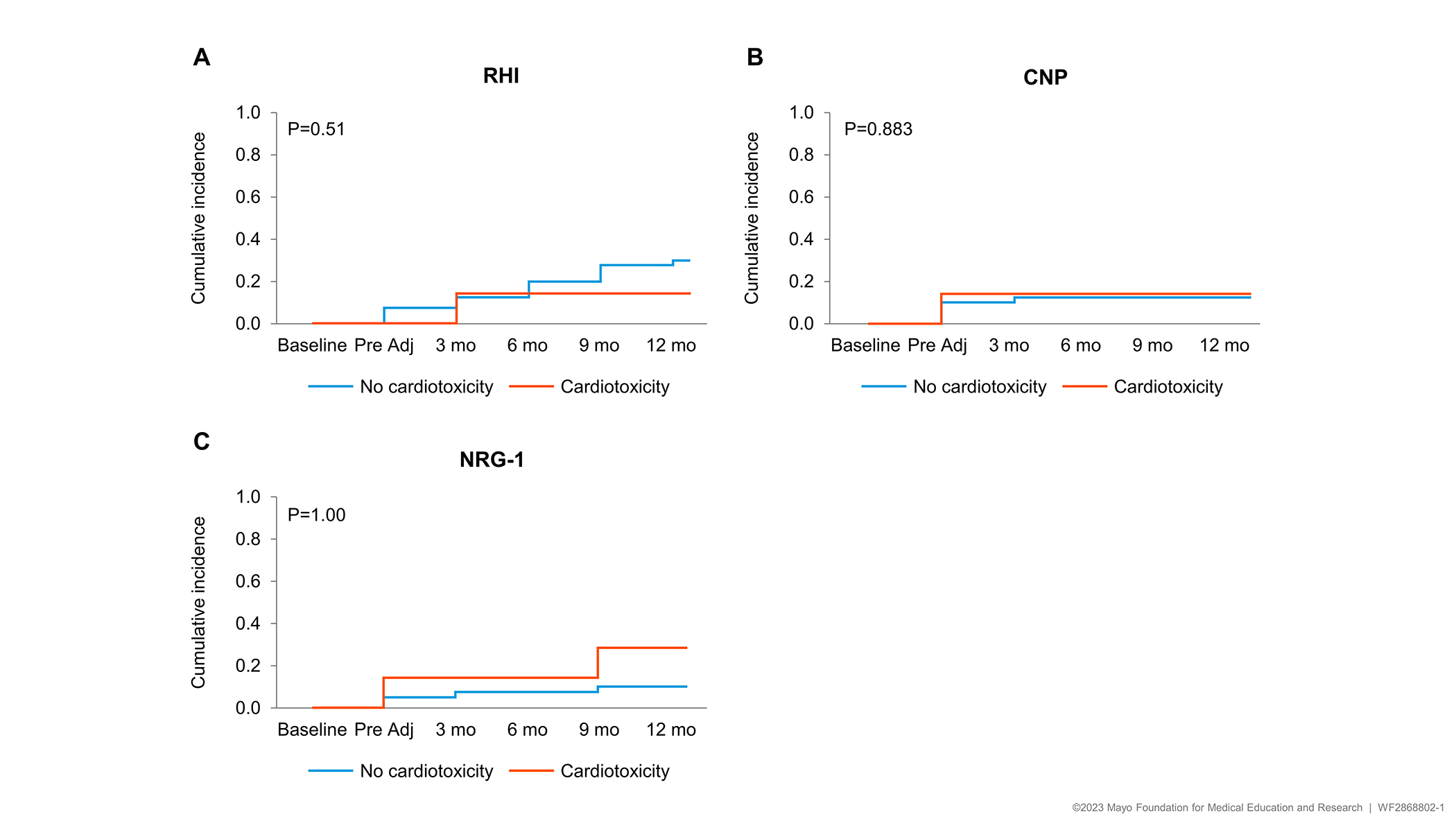


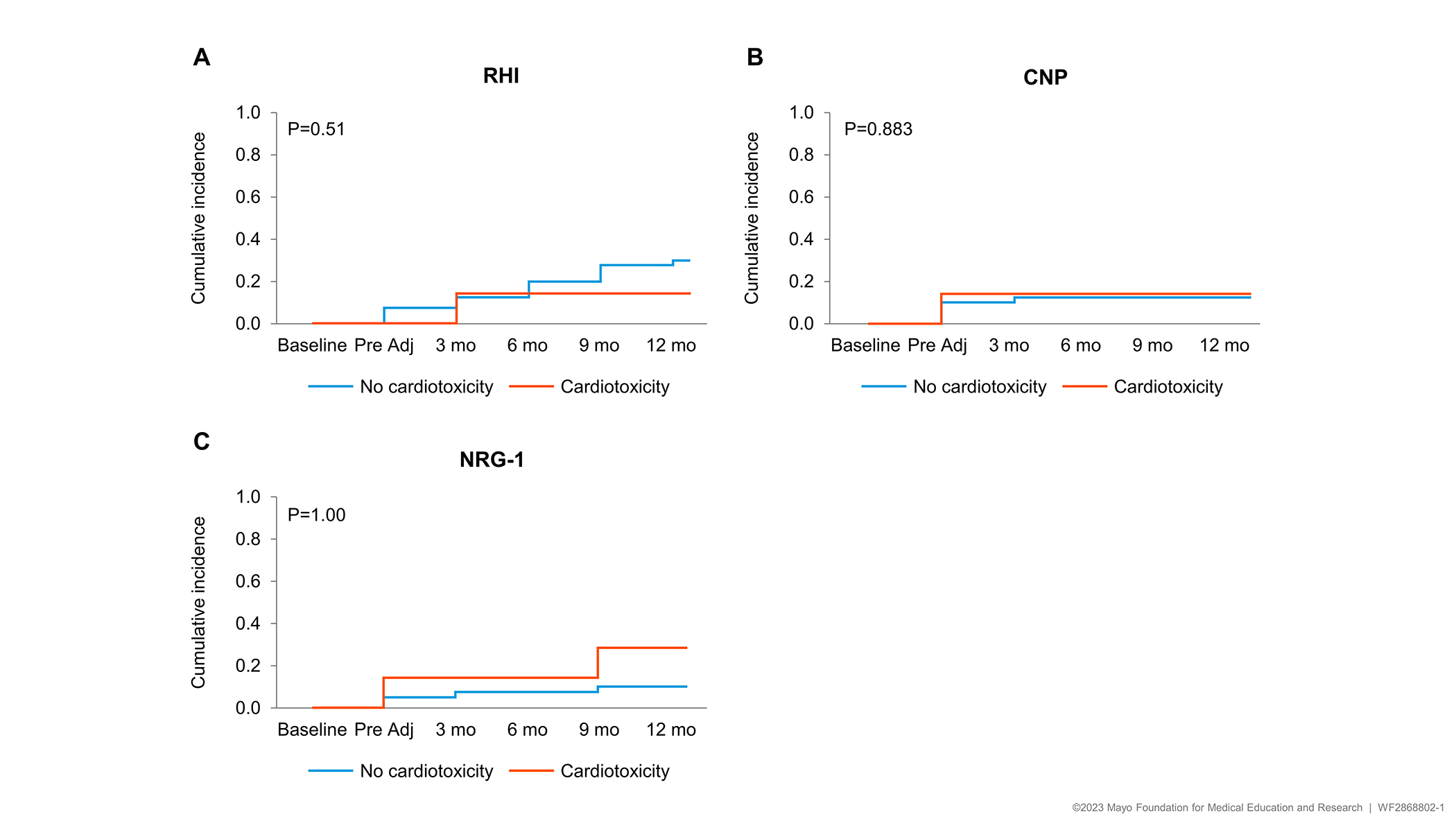


C

B


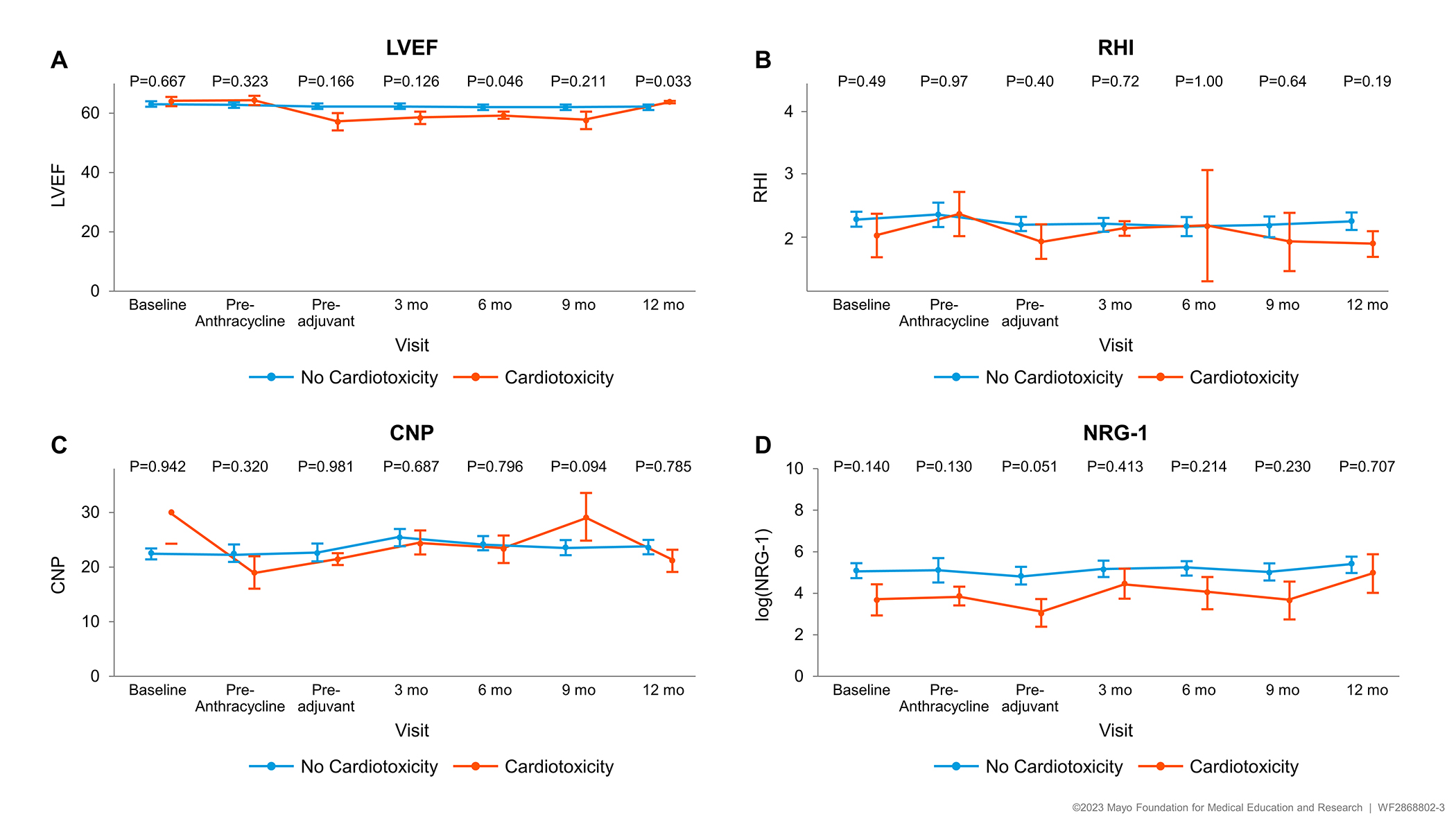


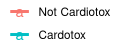

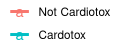

Supplement: oead130_Supplementary_Data [file oead130_supplementary_data.docx]
